# Two new species of *Neoperla* (Plecoptera, Perlidae) from China

**DOI:** 10.3897/zookeys.290.4568

**Published:** 2013-04-16

**Authors:** Li Wei-Hai, Wang Guo-Quan, Qin Xue-Feng

**Affiliations:** 1 Department of Plant Protection, Henan Institute of Science and Technology, Xinxiang, Henan 453003, China; 2 Department of Plant Protection, Guangxi University, Nanning, Guangxi 530004, China

**Keywords:** Plecoptera, Perlidae, *Neoperla*, new species, China

## Abstract

Two species of the genus *Neoperla* from China are described as new: *Neoperla furcostyla* **sp. n.**, and *Neoperla similidella* **sp. n.** The new species are compared to similar taxa.

## Introduction

The stonefly genus *Neoperla* belongs to the family Perlidae and it is the most speciose genus within the subfamily Perlinae (DeWalt et al. 2012). Itis distinguished from other genera of the subfamily by two close located ocelli, and by the abdominal tergum 7 with lobe-like processes and aedeagal tube variously armed with spines or spiny lobes (Sivec et al. 1988). There are up to 68 known species in China described by Chu (1929), Du (1999, 2000a, b), Du and Sivec (2004, 2005), Du and Wang (2005, 2007), Du et al. (1999), Du et al. (2001), Sivec and Zwick (1987), Wu (1935, 1938, 1948, 1962, 1973), Wu and Claassen (1934), Yang and Yang (1990, 1991), Yang and Yang (1992, 1993, 1995a, b, 1996, 1998), Li et al. (2011), Li et al. (2011), Li and Wang (2011), Li et al. (2012) and Li et al. (2012).

In the present paper, we describe two additional species as new to science: *Neoperla furcostyla* sp. n., and *Neoperla similidella* sp. n. from Guangxi autonomous region and Fujian Province, respectively. All types, including paratypes, are deposited in the Entomological Museum of China Agricultural University (CAU). Aedeagi were everted using the cold maceration technique of Zwick (1983).

## Taxonomy

### Neoperla furcostyla

Li & Qin sp. n.

urn:lsid:zoobank.org:act:757404FC-9474-4C97-AAF2-36DB91DB9525

http://species-id.net/wiki/Neoperla_furcostyla

Figs 1 - 2

#### Type material.

Holotype: male, China: Guangxi autonomous region, Nanning City, Wuming County, Mt. Damingshan, 23.4047N, 108.4772E, 9 Aug. 2011, Zhang Ting–Ting. Paratypes: 5 males, same data as holotype.

#### Description.

**Figure 1.**
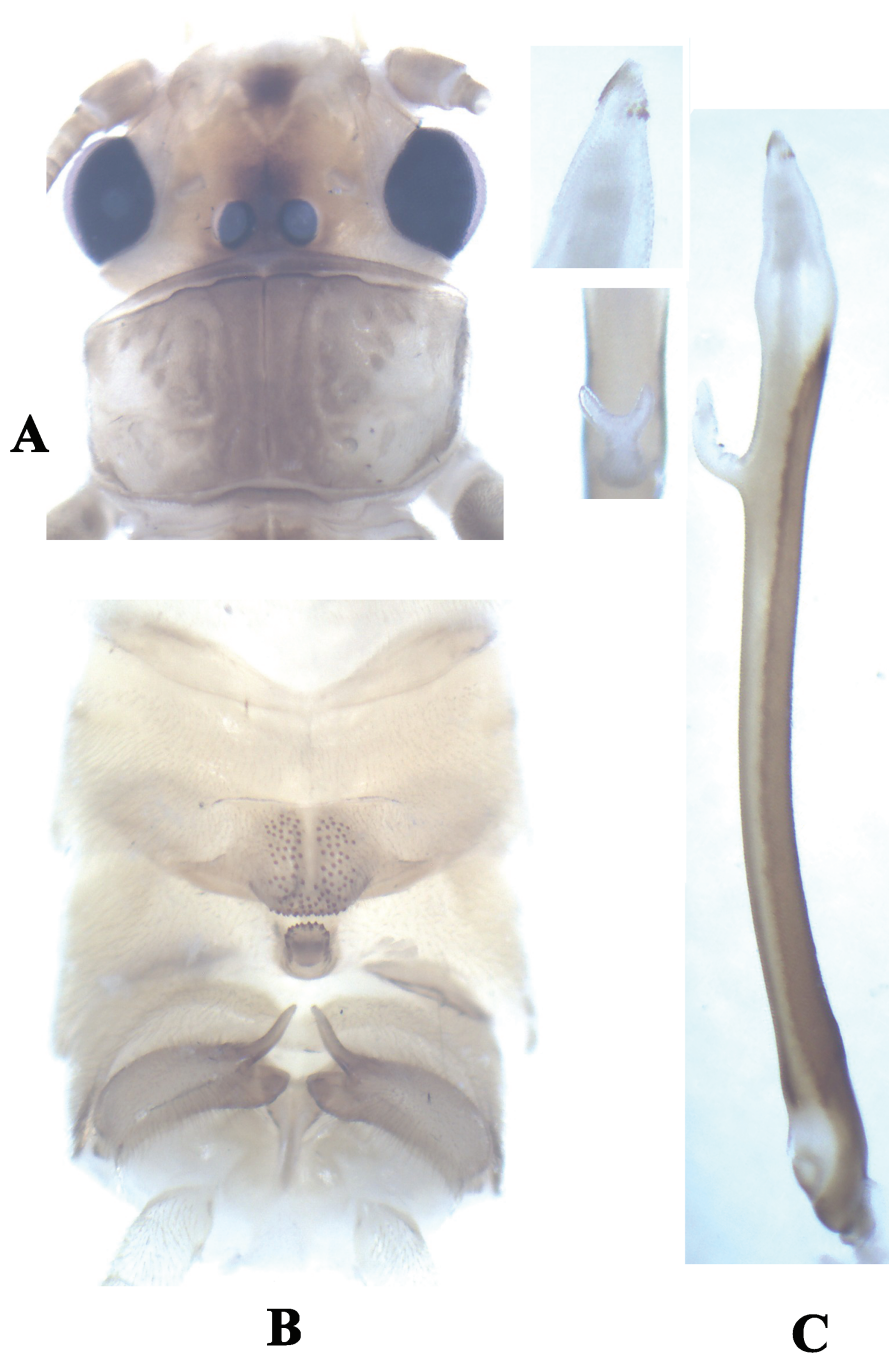
*Neoperla furcostyla* Li and Qin,sp. n. (male). **A** Head and pronotum, dorsal view **B** Terminalia, dorsal view **C** Aedeagus, lateral view.

**Male**. Forewing length 16.6**–**16.9 mm. General body color brownish. Distance between ocelli about as wide as diameter of ocellus. Head slightly wider than pronotum, lateral margins and M-line pale, a large medial portion brown with area between ocelli and a triangular patch on frons darker (Fig. 1A); compound eyes dark; antennae brown. Pronotum pale brown with medial portion brown (Fig. 1A); wing membrane subhyaline, veins brown; legs evenly brown. Abdomen brown, hemiterga darker.

**Figure 2.**
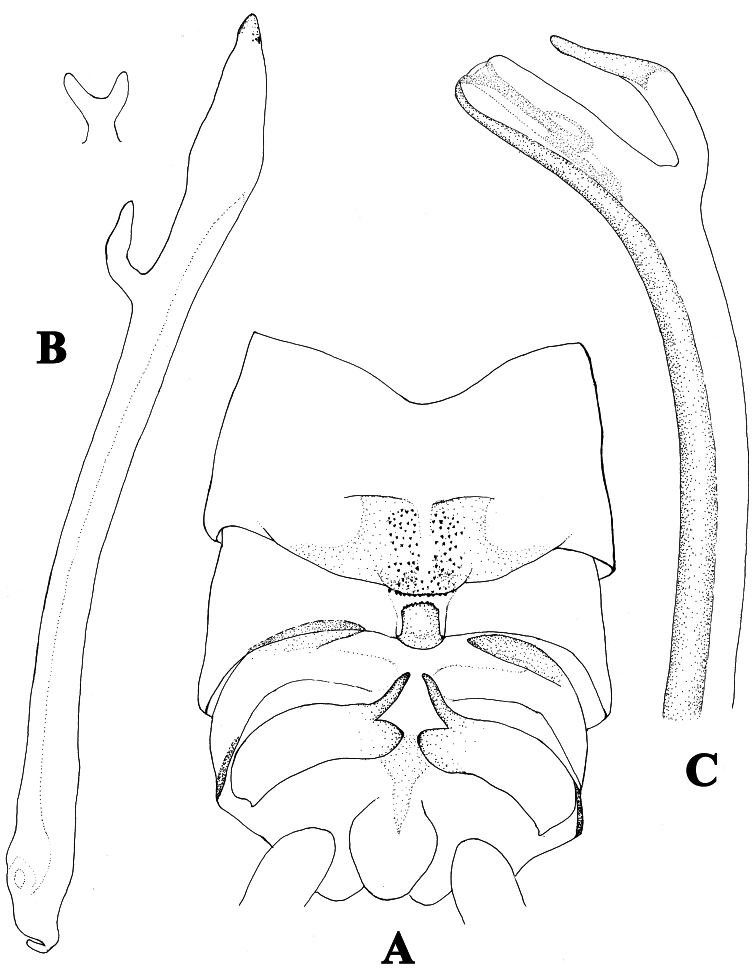
**A–C** *Neoperla furcostyla* Li and Qin,sp. n. (male). **A** Terminalia, dorsal view **B** Aedeagus, lateral view **C** Aedeagus of *Neoperla forcipata* Yang and Yang, lateral view.

#### Terminalia.

Tergum 7 process forming a sclerotized, upraised plateau, covered with many small sensilla basiconica (Figs 1B, 2A). Tergum 8 with a recurved quadrate process bearing small spines at distal margin. Tergum 9 without sensilla patches. Hemitergal processes of tergum 10 short and slightly curved (Figs 1B, 2A). Aedeagal tube very long and almost straight, but bearing a pale bifurcate structure with common stem located subapically (Figs 1C, 2B); dorsal surface heavily sclerotized and membraneous areas on ventral surface gradually enlarged toward tip of the tube. Aedeagal sac very short, about one fifth as long as tube, triangular in shape; with granules around sac apex and several dorsoapical spines and patch of smaller ventroapical spines (Fig. 2B).

#### Female.

Unknown.

#### Etymology.

The specific epithet refers to the subapical forkof the aedeagal tube.

#### Distribution.

China (Guangxi).

**Diagnosis.**The male of *Neoperla furcostyla* is characterized by an elongate, almost straight aedeagal tube bearing a subapical fork with common stem. The aedeagal sac is triangular in shape and barely one fifth as long as tube; several dorsoapical spines and patch of smaller ventroapical spines are present at apex (Figs 1C, 2B). The type of the aedeagus of the new species is also found in *Neoperla forcipata* Yang & Yang, 1992 known from Mt. Wulingshan of Hunan Province of China, but the aedeagus of this species is different. In *Neoperla forcipata* (Fig. 2C), the aedeagal tube is more robust and has an obtuse curve near the subapical fork, and the fork is much larger than that of *Neoperla furcostyla*. The sac of *Neoperla forcipata* though not everted, has an evident lateral flap subapically that is absent on the sac found in *Neoperla furcostyla*. The new species is assigned to the Diehli subgroup of the Montivaga species group (Zwick 1983).

#### Notes.

The holotype of *Neoperla forcipata* Yang and Yang is apparently damaged at the base of the aedeagal tube, very possibly due to an overlooking of this elongate type of tube during the course of dissection. In this case, it is safe to cut the abdominal at the fifth or preceding segments in order to keep this kind of tube intact after dissection.

### Neoperla similidella

Li & Wang sp. n.

urn:lsid:zoobank.org:act:2549F9C7-9FEF-4BF6-B044-783CA798F6F5

http://species-id.net/wiki/Neoperla_similidella

Figs 3 - 4

#### Type material.

Holotype: male, China: Fujian Province, Mt. Wuyishan, Kekao Station, 735 m, 27.7478N, 117.6831E, light trap, 12 Jul. 2009, Shi Li and Liu Xiao-Yan. Paratypes: 2 males, same data as holotype.

#### Description.

**Figure 3.**
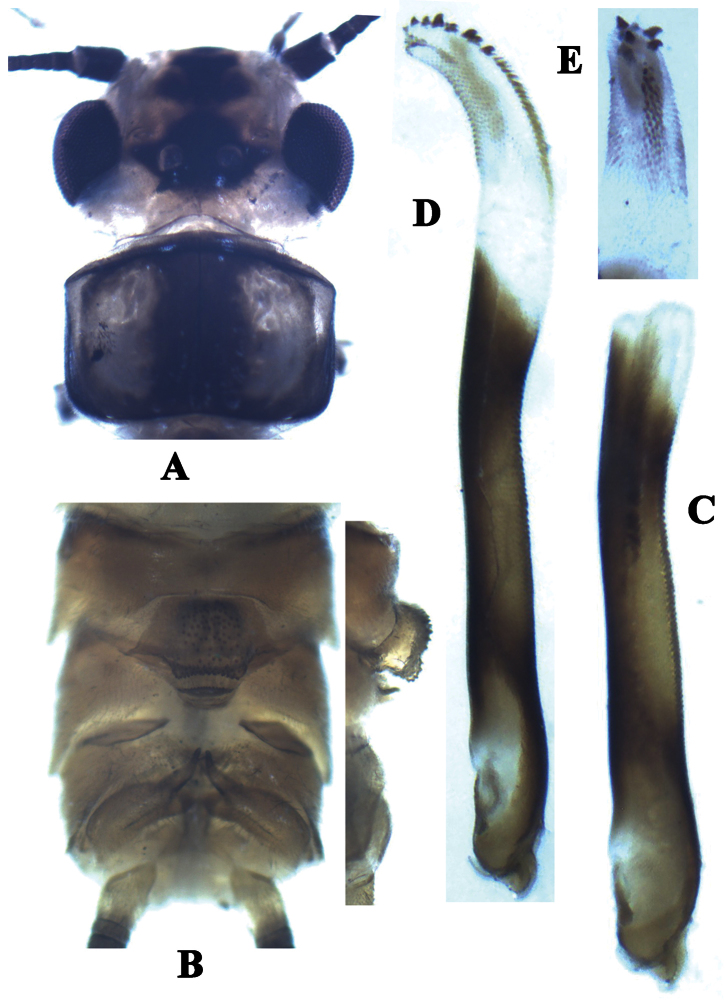
*Neoperla similidella* Li and Wang, sp. n. (male). **A** Head and pronotum, dorsal view **B** Terminalia, dorsal view **C** Aedeagus before eversion, lateral view **D** Aedeagus, lateral view **E** Aedeagal sac, dorsal view.

**Male**. Forewing length 12.6–12.8. General body color dark brown. Distance between ocelli ca. 1.5X as wide as diameter of ocellus. Head slightly wider than pronotum, with a large black ocellar patch barely touching the compound eyes and a black trapezoidal patch on frons (Fig. 3A); compound eyes dark; antennae dark brown. Pronotum dark brown with pale brown to brown lateral margins (Fig. 3A); wing membrane pale brown, veins dark; legs yellow, basal third of tibiae darker. Abdomen dark brown, terminalia darker. Cerci dark except 1^st^ segment brown.

**Figure 4.**
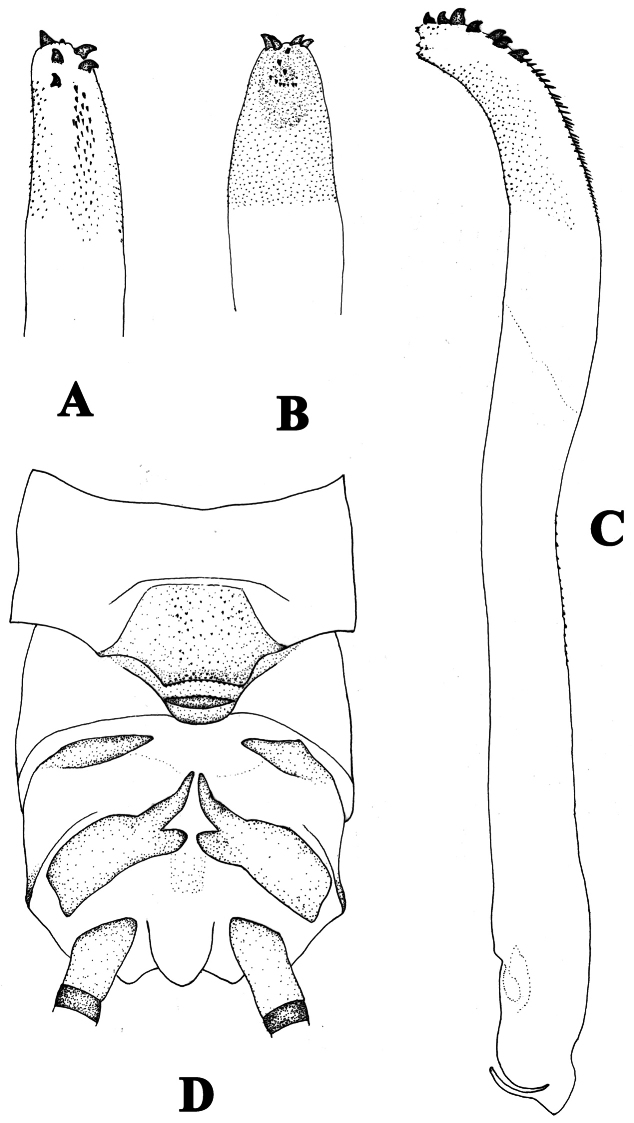
*Neoperla similidella* Li and Wang, sp. n. (male). **A** Aedeagal sac, dorsal view **B** Aedeagal sac, ventral view **C** Aedeagus, lateral view **D** Terminalia, dorsal view.

#### Terminalia.

Process of tergum 7 large, rounded and upraised, mostly covered with sparse sensilla basiconica but margined with denser sensilla basiconica patches (Fig. 3B). Tergum 8 with an upcurved tongue-shaped process, fringed with small distal spines. Tergum 9 without sensilla patches. Hemitergal processes of tergum 10 slightly curved medially (Figs 3B, 4D). Aedeagal tube darkly sclerotized, slender and mostly straight but with a gradual dorsoapical curve, dorsal surface heavily sclerotized, with many spinules on dorsal surface (Fig. 3C). Aedeagal sac about half as long as tube and gradually curved ventrad; along dorsoapical surface with a patch of small and median sized spines, and two rows of large stout spines apically (ca. 7 spines) (Figs 3E, 4A); mostly ventral and lateral surfaces of apical half of sac with small spines (Figs 3D, 4C); apex of the sac with several medium sized spines (Figs 4B).

#### Female.

Unknown.

#### Etymology.

The specific epithet refers to the great similarity to the species *Neoperla idella* Stark & Sivec, 2008.

#### Distribution.

China (Fujian Province).

#### Diagnosis.

This species is characterized by its dark body color and the head with a large black ocellar patch barely touching the compound eyes and a black trapezoidal patch on frons. The aedeagal sac has a patch of small and medium sized spines, and two rows of large stout spines that range from mid length to the apex of the dorsal surface (ca. 7); ventral and lateral surfaces of the apical half of the sac have small spines and the apex of the sac has several medium-sized spines. The aedeagus of the new species is very similar to that of *Neoperla idella*, however in that species the aedeagal tube lacks a subtle dorsoapical curve and the sac is more strongly curved ventrad (Stark and Sivec 2008, figs 36–37). In addition, the aedeagal sac of *Neoperla idella* lacks a ventroapical patch of small spines and also lacks medium sized spines. Both species bear similar patches of large spines on the dorsoapical margin of the sac but in the new species the ventrolateral patch of small and medium spines is located nearer the sac apex than in *Neoperla idella*.

## Supplementary Material

XML Treatment for Neoperla furcostyla

XML Treatment for Neoperla similidella
